# Effect of polybrominated diphenyl ether (PBDE) treatment on the composition and function of the bacterial community in the sponge *Haliclona cymaeformis*

**DOI:** 10.3389/fmicb.2014.00799

**Published:** 2015-01-14

**Authors:** Ren-Mao Tian, On On Lee, Yong Wang, Lin Cai, Salim Bougouffa, Jill Man Ying Chiu, Rudolf Shiu Sun Wu, Pei-Yuan Qian

**Affiliations:** ^1^Divison of Life Science, The Hong Kong University of Science and TechnologyHong Kong, China; ^2^Department of Biology, Hong Kong Baptist UniversityHong Kong, China; ^3^School of Biological Sciences, The University of Hong KongHong Kong, China; ^4^State Key Laboratory of Marine Pollution, The City University of Hong KongHong Kong, China

**Keywords:** PBDEs, sponge-associated bacterial community, 16S rRNA gene pyrosequencing, metagenomics, symbionts

## Abstract

Marine sponges play important roles in benthic environments and are sensitive to environmental stresses. Polybrominated diphenyl ethers (PBDEs) have been widely used as flame retardants since the 1970s and are cytotoxic and genotoxic to organisms. In the present study, we studied the short-period effect of PBDE-47 (2,2′,4,4′-tetrabromodiphenyl ether) treatment on the community structure and functional gene composition of the bacterial community inhabiting the marine sponge *Haliclona cymaeformis*. Our results showed that the bacterial community shifted from an autotrophic bacteria-dominated community to a heterotrophic bacteria-dominated community in response to PBDE-47 in a time- and concentration-dependent manner. A potentially symbiotic sulfur-oxidizing bacterium (SOB) was dominant (>80% in abundance) in the untreated sponge. However, exposure to a high concentration (1 μg/L) of PBDE-47 caused a substantial decrease in the potential symbiont and an enrichment of heterotrophic bacteria like *Clostridium*. A metagenomic analysis showed a selective effect of the high concentration treatment on the functional gene composition of the enriched heterotrophic bacteria, revealing an enrichment for the functions responsible for DNA repair, multidrug efflux pumping, and bacterial chemotaxis and motility. This study demonstrated that PBDE-47 induced a shift in the composition of the community and functional genes in the sponge-associated bacterial community, revealing the selective effect of PBDE-47 treatment on the functions of the bacterial community in the microenvironment of the sponge.

## Introduction

Marine sponges arose 600 million years ago and are the most primitive metazoans found worldwide (Li et al., 1998). They constitute a major component of benthic communities in marine environments and play an essential role in the marine ecosystem in terms of pelagic processes (Dayton et al., 1974; Dayton, 1989) such as the food chain and silicon cycle. Interestingly, marine sponge can form close associations with the symbiotic microbial communities within their bodies (Taylor et al., 2007), and thus, the symbiotic microbial community is sponge-specific and distinct from those in the surrounding seawater and other habitats (Taylor et al., 2007; Lee et al., 2011). Symbiotic microorganisms play essential roles in the sponge body, participating in carbon, nitrogen, and sulfur cycles (Taylor et al., 2007).

Sponge-microbe consortia are sensitive to environmental stresses such as metal pollutants and heat stresses. Recent studies have shown that the sponge-associated microbial community composition can be shifted in response to heat stress and copper treatment (Webster et al., 2001, 2008; Fan et al., 2013; Tian et al., 2014b). In a case study, the bacterial community of the sponge *Rhopaloeides odorabile* was dominated by *Proteobacteria, Actinobacteria, Nitrospira, Acidobacteria* and *Chloroflexi*, whereas at a high temperature, the main population changed to *Proteobacteria, Bacteroidetes* and *Firmicutes*, some of which are pathogenic (Webster et al., 2008). In a second case study, copper treatment resulted in morphological changes in the associated microbes of *R. odorabile*. Moreover, the copper treatment shifted the bacterial community composition, resulting in an enrichment for phylogenetically distant bacterial species (Webster et al., 2001).

Since the 1970s, polybrominated diphenyl ethers (PBDEs) have been widely used as flame retardants in plastics, textiles, polyurethane foam and electrical and electronic appliances, and thus, they have become ubiquitous in some environments. The presence of 22 PBDE congeners in cod, ringed seal, polar bear, and beluga whale in the Arctic have been detected during the last decade (Wolkers et al., 2004). Severely contaminated sediment in the San Francisco Bay could harbor PBDEs at concentrations up to 212 ng/g (Shaw and Kannan, 2009). A study examining blubber biopsy samples from whales raised high concerns that PBDEs may be transferred along the marine food chain, posing a threat to marine mammals, especially to prominent predators (Rayne et al., 2004).

The harmful effects of PBDEs have been documented in marine invertebrates such as polychaetes, gastropods and barnacles in terms of morphology, growth and settlement (Lam et al., 2010; Chiu et al., 2012). In addition, studies have shown that PBDEs can disrupt the levels and the balance of sex and thyroid hormones in vertebrates (Tomy et al., 2004; Han et al., 2011). The cytotoxic and genotoxic effects of PBDE-47 have been demonstrated *in vitro* in human neuroblastoma (SH-SY5Y) cells. In those studies, PBDE-47 inhibited cell viability, enhanced lactate dehydrogenase leakage, and induced cell apoptosis (Zhang and Huang, 2005; He et al., 2008; Yan et al., 2011). PBDE-209 (decabromodiphenyl ether) was also found to be anti-proliferative and to induce apoptosis in tumor cells *in vitro* (Zhang and Huang, 2005). Moreover, the genotoxicity of five PBDE congeners was investigated in chicken DT40 cells, including wild type and mutant cell lines that were deficient in DNA repair pathways. It was found that DT40 cells that were deficient in base excision repair and translation DNA synthesis pathways were hypersensitive to tetra-BDEs and hydroxyl-tetra-BDEs (Ji et al., 2011). Other studies have also demonstrated that PBDEs can covalently bind to DNA mediated by quinone metabolites, forming PBDE-DNA adducts with the potential to damage DNA (Zhao et al., 2002).

Whereas previous studies on the toxicity of PBDEs have mainly focused on vertebrates, mussels, and various eukaryotic cell lines, the effects of PBDEs on sponges and associated microbes are unknown. In this study, we investigated the short-period effects of low and high concentration of PBDE-47 (a dominant congener that is potentially more toxic than others) on the community composition and functional gene profile of microbes inhabiting the marine sponge *Haliclona cymaeformis*, to examine how PBDE-47 will shift the bacterial communities and what genetic features will assist the survival of the enriched bacteria under the stress induced by this pollutant.

## Materials and methods

### Collection of sponge species

In the present study, we used the sponge *Haliclona cymaeformis*, which is a commonly found sponge species in Hong Kong. Intact and healthy sponge colonies were collected from shallow water (at a depth of ~1 m) at Po Toi O Village, Hong Kong (22° 16′ 32.96″N, 114° 17′ 39.95″E) and transported back to the laboratory in natural seawater. The sponge colonies were then maintained in an aquarium supplied with circulating sand-filtered seawater and sufficient aeration for 1 week to stabilize the sponges prior to experimentation. Colonies of *H. cymaeformis* comprise independent gemmules (each with one osculum) that characterize the clonal budding mode of reproduction (Figure S1).

### Treatment of the sponge samples with PBDE-47

In this study, we investigated the short-period effects of low (10 ng/L, a typical concentration in polluted seawater) and high (1 μg/L, a representative concentration in seriously polluted areas like sediment) doses of PBDE-47 over 2 and 6 days, respectively (based on preliminary experiments, data not shown). A stock solution of PBDE-47 (>99% purity, Chem Service, Inc., West Chester, PA) at 3 mg/mL was prepared by dissolving the chemical in dimethyl sulfoxide (DMSO), which was then further diluted to 10 ng/L and to 1 μg/L in seawater. A solvent control group (DMSO at 1 μL/L) and negative control group (seawater alone) were used.

Using denaturing gradient gel electrophoresis (DGGE), a preliminary screening of the bacterial community showed highly similar band patterns of the different sponge colonies (data not shown). Therefore, we used one large sponge colony as starting material and cut it into 27 small colonies (each with 2–3 complete gemmules) for the experiments (similarly to a study of sponges exposed to heat stress, Webster et al., 2008). The small colonies were placed in 2-L aquariums supplied with 1.5 L of filtered seawater with the corresponding PBDE-47 concentrations and sampled at 0, 2, and 6 days. Figure S2 provides a detailed description of the treatment. The room temperature was stabilized at 22–25°C to ensure an appropriate water temperature for the sponges.

### DNA extraction of sponge samples

Sponge tissues were sampled on days 0, 2, and 6 of the treatment period. At each time point, three sponge colony replicates from each group were sampled, and intact gemmules of the colonies were cut into small pieces using sterile stainless steel scissors. The samples were further homogenized with a sterilized mortar and pestle to release the microorganisms. The homogenates were centrifuged at 100 × g for 1 min to pellet the debris and fragments of sponge tissues. The supernatant was then filtered through a 12-μm filter membrane (GE Water & Process Technologies). The filtrate of microorganisms was stained with safranin (Bacto Laboratories Pty Ltd.) and observed under a 100 × microscope (Olympus BX51, USA). Most of the filtered microorganisms were ~1 μm in size, indicating that nearly all of the eukaryotic cells had been removed from the filtrate. The filtrate was then centrifuged at 10,000 × g for 5 min, and the pellets, presumably containing the microbial cells, were stored in 800 μL of DNA extraction buffer (500 mM NaCl, 50 mM Tris-HCl [pH 8], 40 mM EDTA, 0.75 M sucrose). DNA extraction was conducted according to a previous protocol (Lee et al., 2009). Briefly, the bacteria were lysed using 10 μL of lysozyme (100 mg/mL), and the protein was digested using 80 μL of 20% SDS and 8 μL of proteinase K (10 μg/μL). DNA was extracted twice with chloroform:isoamyl alcohol (24:1) and then precipitated with 100% isopropanol followed by washing in 75% cold ethanol. The quality and quantity of the extracted DNA were checked using a NanoDrop ND-1000 device (Thermo Fisher, USA) and agarose gel electrophoresis.

### PCR amplification of the 16S rRNA gene for pyrosequencing

The hypervariable V3-V5 region of the 16S rRNA gene was amplified using the primer set 341F (5′ CCTACGGGAGGCAGCAG 3′) and 907R (5′ CCGTCAATTCCTTTRAGTTT 3′). A unique 8-nucleotide barcode was added to both primers for multiplexed pyrosequencing using barcrawl (Pourmand et al., 2002; Frank, 2009). Each 20-μL PCR reaction consisted of 4 μL of 5 × Phusion HF Buffer (M0530S New England BioLabs Inc.), 1.6 μL of dNTPs (2.5 μM each), 1 μL of each primer (10 μM), 0.6 μL of DMSO, 10 ng of template DNA, 0.2 μL of Phusion^®^ High-Fidelity DNA Polymerase (0.4 units) and 10.6 μL of pure water. PCR was performed with a thermal cycler (Bio-Rad, USA) using the following program: an initial denaturation at 98°C for 1 min; 25 cycles at 98°C for 10 s, 60°C for 30 s and 72°C for 20 s; and a final extension at 72°C for 5 min. The PCR products were detected by agarose gel electrophoresis, and the purified concentrations were measured using a NanoDrop ND-1000 device (Thermo Fisher, USA). Equal amounts of the barcoded amplicons were mixed and pyrosequenced using a ROCHE 454 FLX Titanium platform.

### Analysis of the 16S rRNA gene sequences

The pyrosequencing datasets were analyzed using the Quantitative Insights Into Microbial Ecology (QIIME) pipeline (Caporaso et al., 2010b). The sequences were filtered using the following quality controls: sequences with a quality score <25; homopolymers with a length >6, <100, or >1000 bp; >6 ambiguous bp; >3 mismatches in the primer and 1 mismatch in the barcodes. Qualified sequences were sorted into their corresponding samples according to the unique barcodes. The reads were further subjected to a denoising program (using greedy scheme algorithm) for a second round of quality control (Reeder and Knight, 2010). The OTUs were selected at the species level with 3% dissimilarity, and each OTU was assigned using the Ribosomal Database Project (RDP) Classifier (version 2.2) (Wang et al., 2007) with the Silva10.8 database (Pruesse et al., 2007) with a confidence level of 50%. Representative sequences of each OTU were aligned using MUSCLE (Edgar, 2004) and PyNAST (Caporaso et al., 2010a). Chimeric sequences identified by Chimera Slayer (Caporaso et al., 2010a) were removed. Similarities among the bacterial communities were evaluated by UniFrac Principal Coordinates Analysis (PCoA). A UniFrac Jackknife tree was constructed to display the relationships of the bacterial community in each sample. The statistical significance of the pairwise sample dissimilarity was assessed by the *P*-test.

A phylogenetic tree of a potentially symbiotic bacterium (unassigned *Ectothiorhodospiraceae*) was constructed using Mega 6.05 based on the partial 16S rRNA gene and reference sequences from NCBI (hits from the NR/NT database with an identity >95%, which mainly included unculturable bacteria, and hits from the 16S rRNA sequence database with an identity >85%, which included identified species).

### Metagenomic sequencing and analysis

The metagenomic DNA of the two replicates (1 μg each) of sample D6 (DMSO control for the 6-day treatment) and H6 (6-day high-dose treatment) was equally pooled and used for Roche 454 shotgun pyrosequencing. Quality control was conducted for the raw metagenomic DNA sequences using the NGS QC Toolkit (version 2.3) (Patel and Jain, 2012) to remove sequences with an average quality score less than 20, a read length less than 100 bp and homopolymers of more than 6 bp. Sequences containing ambiguous bases of more than 5 bp, including the ambiguous bases, were further trimmed. Artificial Duplicate Reads were removed using the program CD-HIT-454 (Niu et al., 2010). Qualified reads were used for a blast analysis (BLAST 2.2.27) against the COG database (http://string-db.org/; version 9.1). The best hits, with an *E*-value >1e-5, bit score >100 and alignment length >30% of the query, were selected as the functional annotation of the reads. Comparison of the functional profiles of pairwise samples was performed with STAMP (Parks and Beiko, 2010) using the pairwise comparison method and Fisher's exact test followed by Storey FDR correction as previously described (Mason et al., 2012).

### Nucleotide sequence submission

The data for the 16S rRNA amplicon and metagenomic sequencing for the PBDE-47 treatment experiment were submitted to the Sequence Read Archive (SRA) database of NCBI under accession numbers SRP018357 and SRP038119, respectively.

## Results

### PBDE-47 affected sponge-associated bacterial communities in a concentration- and time-dependent manner

After 16S rRNA gene amplicon pyrosequencing, a total of 216,656 raw sequences were obtained, of which 164,747 (average length of 519 bp) were recovered by quality control as clean data for subsequent analysis. Rarefaction analysis based on the number of operational taxonomic units (OTUs) and the Shannon index (Figure S3, Table S1) revealed that the sequencing depth was sufficient and that the overall bacterial communities were well described.

**Figure 1:**
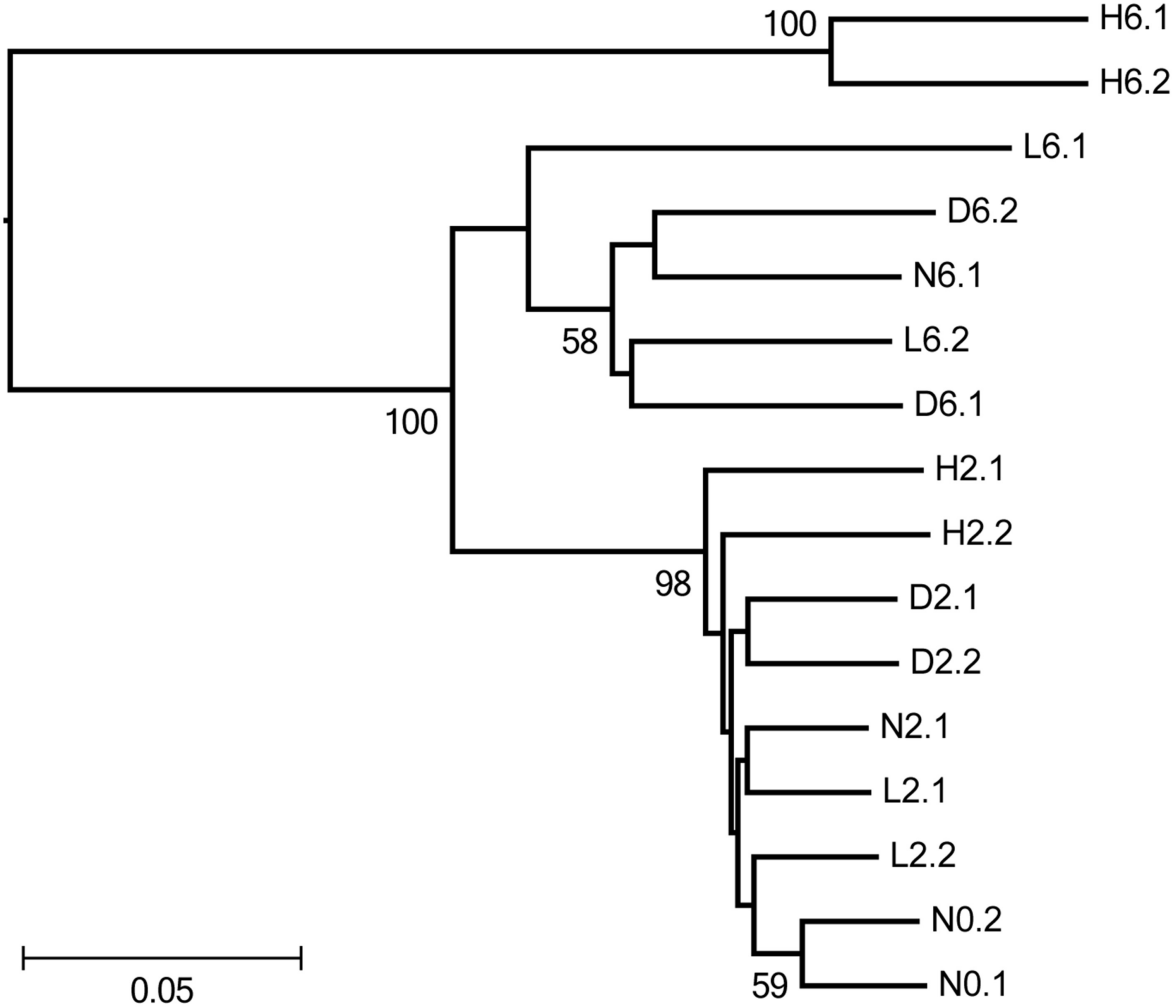
**Jackknifed UPGMA clustering (using the weighted UniFrac metric) showing the similarity of sponge-associated bacterial communities based on pyrosequenced 16S rRNA genes**. Bootstrap values >50 are shown at the nodes. N, D, L, and H represent the negative control, DMSO control, low-dose treatment and high-dose treatment, respectively. The digits following represented days of time point and the last digits represented replicate numbers.

The similarity of the sponge-associated bacterial communities was calculated and displayed using Principal Coordinate Analysis (PCoA, Figure S4) and UPGMA clustering analysis (Figure 1). In the PCoA analysis, PC1, which explained 55% of the observed variations, separated H6 from the remaining samples; PC2 explained 31% of the variations and separated the 6-day samples from the 0- and 2-day samples. H6 was distant in comparison to the other samples, and a pairwise statistical test indicated that the bacterial communities from the two H6 samples (H6.1 and H6.2) were significantly different (*P*-test; *p* < 0.01) from all of the other samples. In the Jackknife UPGMA tree (Figure 1), N0, N2, and L2 clustered together and H2 emerged as an independent group (but with a relatively low dissimilarity). For the 6-day samples, N6, D6, and L6 were in the same cluster and H6 was located a large distance from all of the other samples. Taken together, these results indicated that PBDE-47 shifted the bacterial communities in the sponge in a concentration- and time-dependent manner.

### Proteobacteria decreased while firmicutes were enriched during the treatment

**Figure 2:**
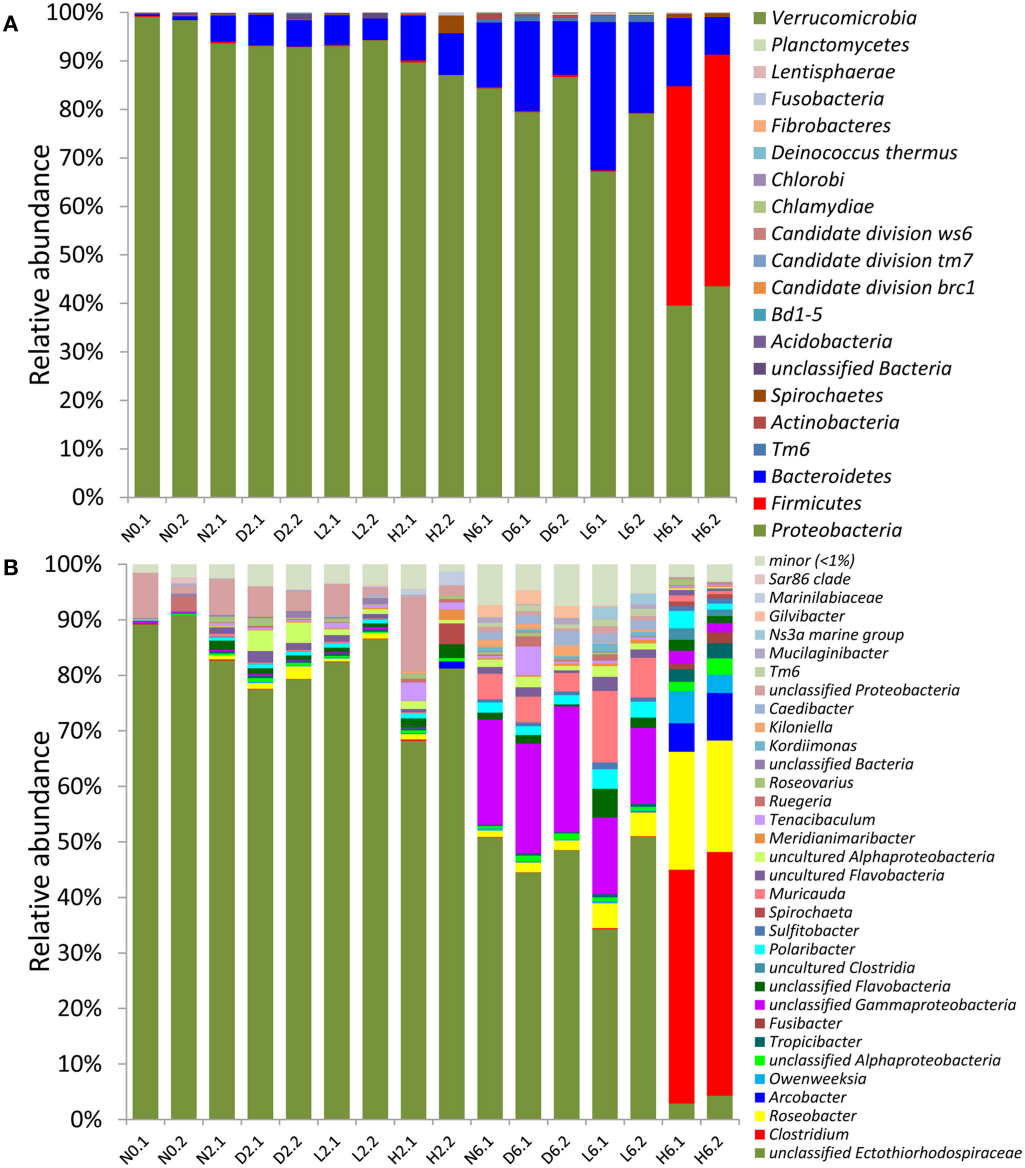
**Taxonomic composition of the bacterial communities in the treated sponge samples and controls (with read numbers >1000)**. Classification of the pyrosequenced reads was performed using the RDP classifier against the Silva10.8 database at the **(A)** phylum and **(B)** genus levels. The minor group refers to genera representing <1% in all samples. Sample abbreviations are provided in Figure 1.

Altogether, 17 phyla were recovered from the sponge bacterial communities (Figure 2A). The untreated samples were dominated by *Proteobacteria* (97.6%). In the 2-day treatment samples, the low- and high-concentration-treated samples (L2 and H2) showed a similar community structure compared with the negative control (N2) and the solvent control (D2), indicating that the treatment had a slight effect within 2 days. In the 6-day samples, however, the *Proteobacteria* decreased from 82.2% (in D6) to 71.4% in L6 and 41.7% in H6. In contrast, the *Firmicutes* was remarkably enriched from <1% in N6, D6 and L6 to 46.4% in H6. The bacterial communities obtained from the DMSO controls were highly similar to those of the negative controls, indicating that DMSO had no obvious effect on the bacterial community. These results showed that PBDE-47 could shift the structure of the sponge-associated bacterial community, resulting in a remarkable decrease in *Proteobacteria* and an enrichment for *Firmicutes*.

### PBDE-47 shifted the potentially symbiotic SOB-dominated community to a heterotroph-dominated community

At the genus level, up to 87.6% of the reads from the untreated sponge at day 0 (N0) were classified as unassigned *Ectothiorhodospiraceae* (Figure 2B). The 16S rRNA gene sequence (524 bp) had a similarity of 96.4% to a sequence from the sponge-specific cluster database (ID: EF159783_SC153) (Simister et al., 2012). A phylogenetic analysis showed that it was located in a sponge-specific cluster (Figure S5), and the most closely related bacteria were *Thioalkalivibrio* (92% identity to *Thioalkalivibrio nitratireducens* DSM 14787). *Ectothiorhodospiraceae* includes numerous thioautotrophic members that can oxidize reduced sulfur and acquire energy during this process. Our genomic analysis indicated that the dominant bacterium (the unassigned *Ectothiorhodospiraceae* in Figure 2) had the capacity to perform sulfur oxidation and CO_2_ fixation, thus playing a potentially symbiotic role by detoxifying sulfide in the host sponge (Tian et al., 2014a).

On day 6, the abundance of the sulfur-oxidizing bacterium (SOB) was significantly reduced from 41.2% in D6 to 3.6% in H6 (*t*-test; *p* < 0.01), while *Clostridium* was significantly enriched (*t*-test; *p* < 0.01) and occupied up to 43% of the community population in H6. Other significantly enriched genera in H6 (*t*-test; *p* < 0.05) included *Roseobacter* (16.3%), *Polaribacter* (3.2%), and *Owenweeksia* (6.0%), which were generally heterotrophic. These results indicated that PBDE-47 shifted the potentially symbiotic autotrophic SOB-dominated community to a heterotrophic community.

### Selective effect of PBDE-47 treatment on the functions of the bacterial community

The metagenomic data for the microbial community of the treated sponge and the control included 426,468 and 441,416 raw reads with an average length of 506 and 507 bp, respectively. After quality control, 310,880 and 278,911 high-quality reads were annotated for the control and the treated sponge. The composition of functional genes in the control and PBDE-47-treated sponge metagenome were then compared.

**Figure 3:**
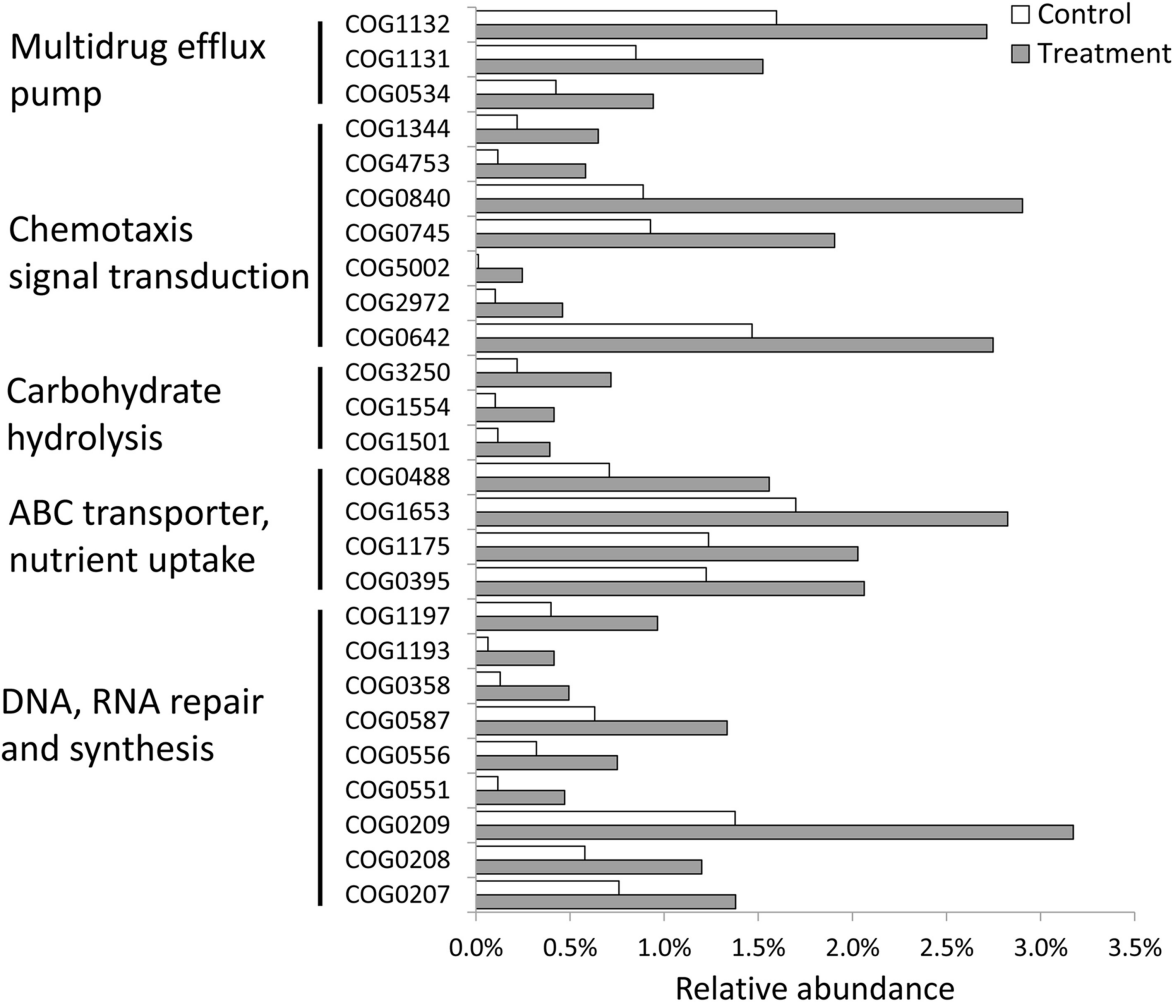
**Significantly changed (Fisher's exact test, *p* < 0.05) functional genes in metagenomes for the control and PBDE-47-treated sponge samples**. The y-axis represents the functional gene category, and the x-axis represents the relative abundance.

In total, there were 12,548 functional categories (COG, KOG, and NOG) in the two metagenomic datasets, of which 108 categories were significantly increased or decreased (Fisher's exact test; *p* < 0.01). Among the 48 significantly increased prokaryotic functional categories, 24 categories were involved in DNA synthesis and repair (including DNA mismatch repair and excision repair systems), multidrug efflux pumps (including the Na^+^ driven pump and ABC-type transporter pump), the ABC transporter for nutrient uptake (including the ABC-type sugar transport permease and periplasmic component), and chemotaxis signal transduction (Figure 3). These results indicated that the PBDE-47 treatment had a selective effect on the functions of the bacterial community in the sponge.

## Discussion

In the present study, we examined the effects of PBDE-47 treatment on the bacterial community structure in the sponge *H. cymaeformis*, and the subsequent response of the community (in terms of the functional gene profile) to the stress induced by PBDE-47. The treatment had a time- and concentration-dependent effect on the bacterial community composition. Low- (10 ng/L) and high (1 μg/L)-dose treatments differed remarkably in terms of the effects on the bacterial community, and a long treatment duration intensified the effect of the high dose.

As described in a study examining heat treatment of the sponge (Webster et al., 2008), the inter-sponge variation was not recovered by DGGE screening. Similarly, in our study, the sponge *Haliclona cymaeformis* displayed extremely low inter-sponge and inter-tissue variations in bacterial community composition. Therefore, we selected a large parental sponge and generated small individual colonies for the PBDE-47 treatments. The results showed remarkable variations in the bacterial community caused by the high-dose treatments compared to the controls and low-dose treatments.

The high dose of PBDE-47 coupled with a long duration (6 days) of treatment substantially decreased the abundance of the unclassified *Ectothiorhodospiraceae*, which is a potentially symbiotic SOB in the sponge. Sulfur metabolism has been described in other sponges in which sulfur-reducing bacteria (SRB) generate sulfide (sulfate as the electronic acceptor) and SOB oxidize sulfide (Hoffmann et al., 2005; Taylor et al., 2007) and generate elemental sulfur and sulfate. In the present study, autotrophic SOB were dominant in the sponge *H. cymaeformis* and might play a symbiotic role by oxidizing sulfide as described in our previous study (Tian et al., 2014a). However, we showed that the SOB in sponge were remarkably decreased by the high-dose treatment with PBDE-47, which might disturb normal sulfur cycling and the symbiosis between the bacterium and the sponge host. The remarkable decrease in the dominant SOB by PBDE-47 treatment was similar to the effect of copper treatment observed in our previous study of the same sponge species (Tian et al., 2014b). Although there was no apparent necrosis in the PBDE-47-treated sponge samples, the color of the colonies became darker green in comparison to the controls, suggesting an effect of the treatment on the sponge-microbe consortium.

In contrast, the *Clostridium* and other heterotrophs were highly enriched in the community. A phylogenetic analysis (data not shown) revealed that the *Clostridium* in this study was located within the clusters of heterotrophic soil bacteria, including *C. aestuarii*, *C. carnis*, and *C. sardiniense*, suggesting a heterotrophic lifestyle of the *Clostridium* species in this study. The taxonomic analysis of the enriched functional genes showed that *Clostridium* is the dominant lineage in the taxonomic assignment (data not shown), which suggested that those beneficial genetic features assisted *Clostridium* survive under the stress of PBDE-47 treatment. Recent studies on the response of the microbial community to environmental stresses also showed a similar trend toward an enrichment of heterotrophs such as *Firmicutes* and *Bacteroidetes* (Webster et al., 2008; Tian et al., 2014b).

In terms of toxicity, PBDEs have been demonstrated to have harmful effects on the structure of DNA, and potential mechanisms underlying this effect have been documented in several studies (Zhao et al., 2002; He et al., 2008; Ji et al., 2011; Yan et al., 2011). PBDEs can covalently bind to DNA via quinone metabolites, resulting in the formation of a DNA-PBDE adduct. PBDEs can further cause DNA breakage and fragmentation. In addition to genotoxicity, PBDE-47 has also been shown to be cytotoxic, impacting cell viability (Zhang and Huang, 2005; He et al., 2008).

**Figure 4:**
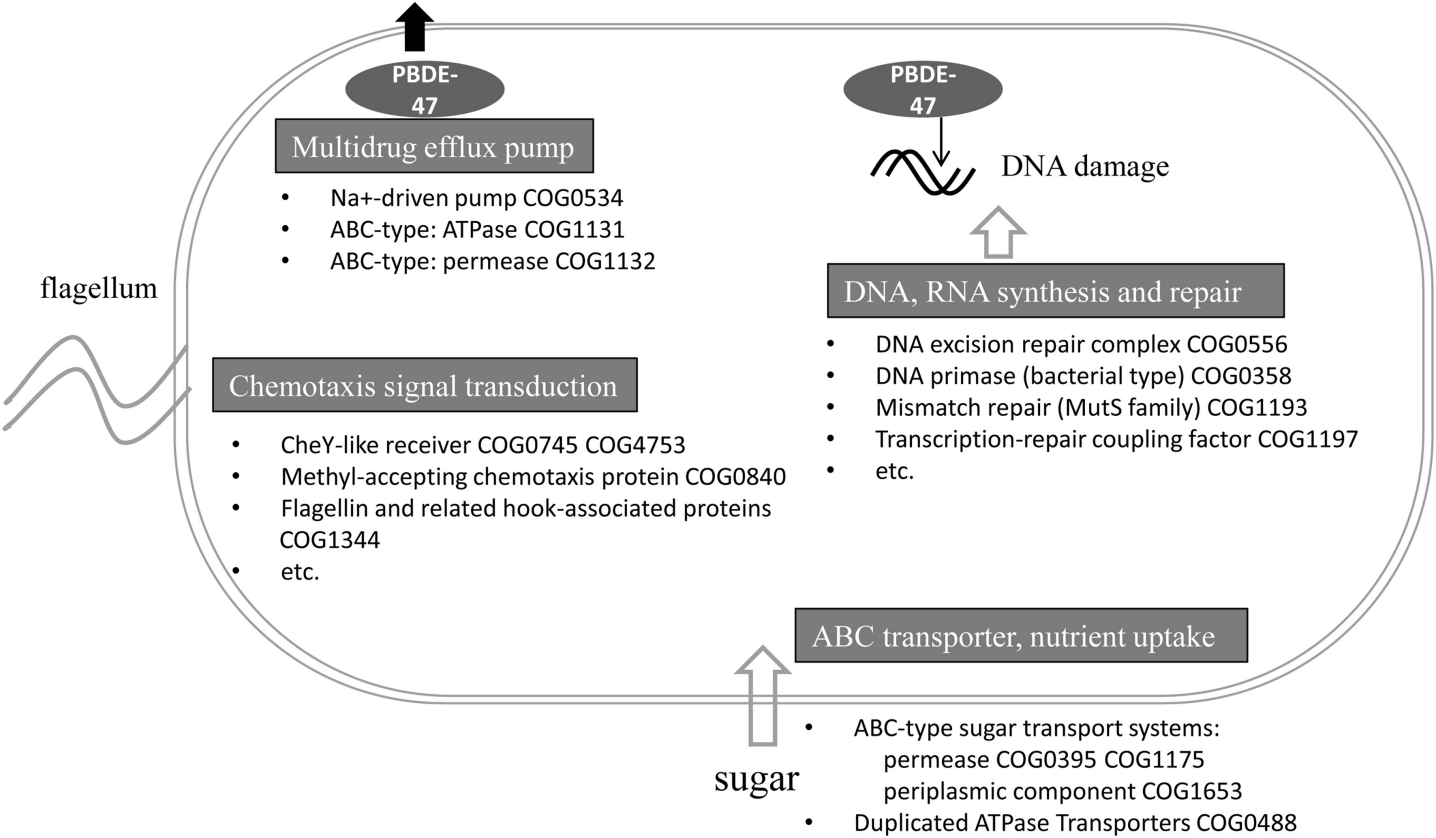
**Schematic showing potential mechanisms used by the bacterial community to survive the stress caused by PBDE-47**. Functional genes (with the COG number) involved in DNA and RNA synthesis and repair, multidrug efflux pumps, the ABC transporter, nutrient uptake and chemotaxis signal transduction are shown.

In the present study, the functional gene composition of the sponge-associated bacterial community was affected under PBDE-47-induced stress. Exposure to PBDE-47 selected for a set of functions that may facilitate survival during the stress caused by PBDE-47 (Figure 4). In our treatment experiment, the enriched functional genes related to DNA repair and synthesis might function to repair the DNA damage caused by PBDE-47; multidrug efflux pumps might function to transport the harmful PBDE-47 out of the bacterial cells; and chemotaxis genes might serve to guide bacteria to zones of minimal PBDE-47 concentration in the microenvironment of the sponge body. The selective effect of the treatment on the bacterial function suggested a survival mechanism of the bacterial community under the stress of PBDE-47.

In contrast to the effect of copper treatment (enriching for functions related to bacterial motility and chemotaxis, bacterial capsule synthesis, virulence, and bacterial signaling and regulation) on the same sponge species (Tian et al., 2014b), different functions (excluding bacterial motility and chemotaxis) such as DNA repair and multidrug efflux pumps were enriched by the PBDE-47 treatment, demonstrating that the toxicity and stress exerted by PBDE-47 on the bacterial community differs from that produced by copper.

### Conflict of interest statement

The authors declare that the research was conducted in the absence of any commercial or financial relationships that could be construed as a potential conflict of interest.
