# Supplementary material for: Effect of polybrominated diphenyl ether (PBDE) treatment on the composition and function of the bacterial community in the sponge Haliclona cymaeformis
Source: Front Microbiol. 2015 Jan 14;5:799. doi: 10.3389/fmicb.2014.00799 (PMC4294214; doi:10.3389/fmicb.2014.00799)
Supplement: Supplementary file 1 [file DataSheet1.DOCX]

**. Supplementary materials**

**Table S1.** Sponge sample names, number of sequences acquired and number of OTUs at 3% dissimilarity. The Shannon index represents the diversity of each sample (subsample size: 3000). N6.2 was missing due to the impaired health of this replicate. A blank space indicates that the results were not applicable due to an insufficient number of reads (<3000).

| Sample | Sample ID |  | No. of reads | No. of OTUs | Shannon index |
| --- | --- | --- | --- | --- | --- |
| Untreated sponge at day 0 | NO.1 |  | 10989 | 115 | 1.86 |
|  | NO.2 |  | - | - | - |
| Untreated sponge at day 2 | N2.1 |  | 7958 | 178 | 2.47 |
|  | N2.2 |  | - | - | - |
| DMSO control at day 2 | D2.1 |  | 22235 | 321 | 2.81 |
|  | D2.2 |  | 18357 | 257 | 2.49 |
| Low-dose treatment for 2 days | L2.1 |  | 5381 | 145 | 2.58 |
|  | L2.2 |  | 6546 | 147 | 1.97 |
| High-dose treatment for 2 days | H2.1 |  | 4140 | 160 | 3.22 |
|  | H2.2 |  | - | - | - |
| Untreated sponge at day 6 | N6.1 |  | 5790 | 230 | 4.19 |
| DMSO control at day 6 | D6.1 |  | 12123 | 284 | 4.45 |
|  | D6.2 |  | 12919 | 317 | 4.24 |
| Low-dose treatment for 6 days | L6.1 |  | 3706 | 208 | 4.95 |
|  | L6.2 |  | 4792 | 189 | 3.92 |
| High-dose treatment for 6 days | H6.1 |  | 11875 | 230 | 4.05 |
|  | H6.2 |  | 18295 | 295 | 4.02 |

**
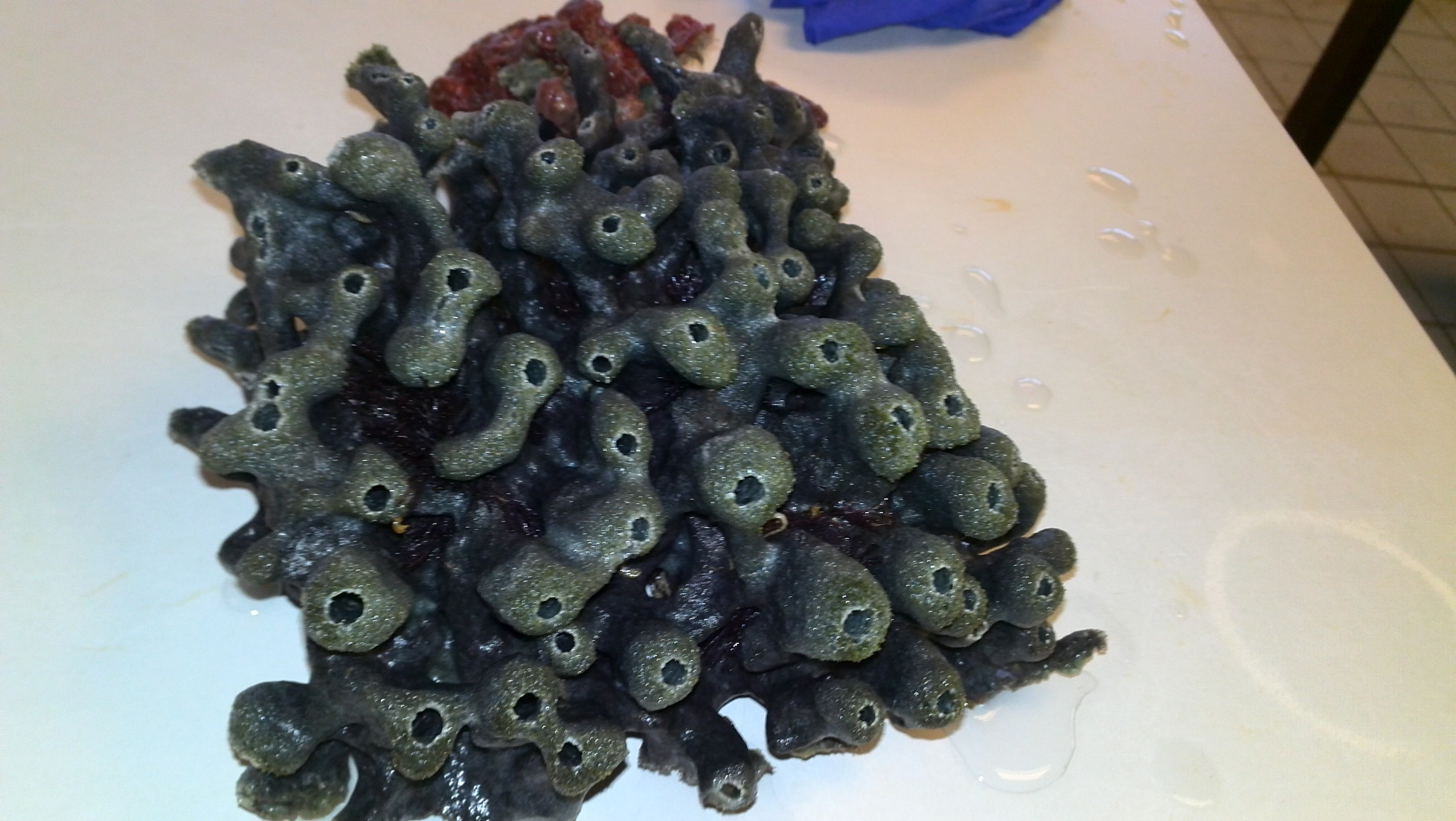
**

**Figure S1.** The sponge species *Haliclona cymaeformis* used in this study. The sponge consists of relatively independent gemmules (each with one osculum) resulting from asexual reproduction. More information about the morphology, physiology and ecology of this sponge can be accessed at <http://www.gbri.org.au/SpeciesList/Haliclonacymaeformis%7CRonjaSchmidt.aspx?PageContentID=4050>.

**Figure S2.** Schematic showing the different treatment concentrations (low: 10 ng/L, high: 1 µg/L) and sampling time points (0, 2 and 6 days). The sponge colonies were maintained in water tanks with running seawater and aeration for 7 days for stabilization. One large colony was cut into 27 small colonies that were maintained in the same tank for one day prior to the treatment. The small colonies in triplicate were placed in the corresponding aquaria and sampled at 0, 2 and 6 days.

**
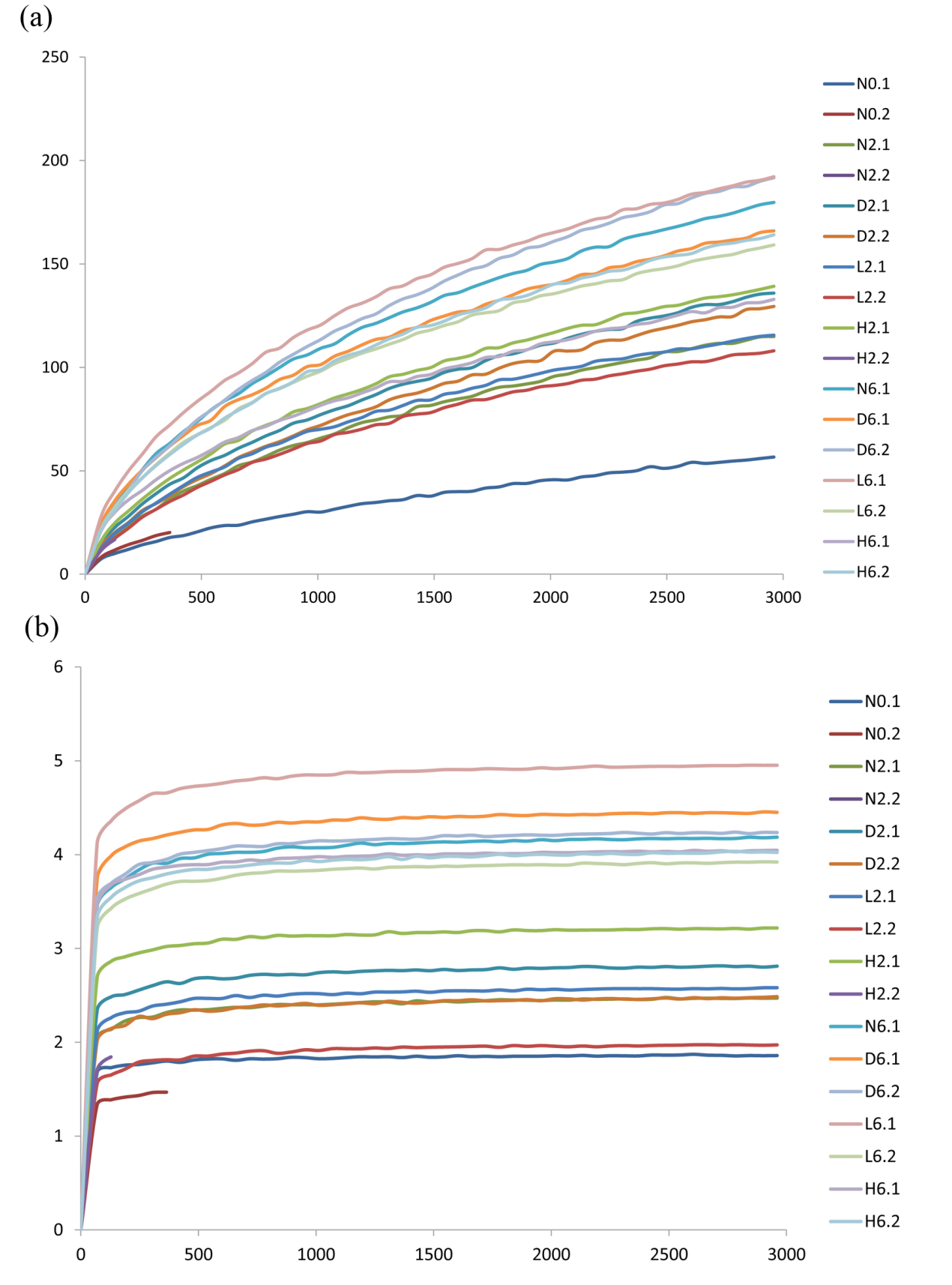
**

**Figure S3.** Diversity of bacterial communities in the sponge samples. Rarefaction curve of the Shannon index based on bacterial OTUs at a dissimilarity level of 3%, showing the diversity of the bacterial communities in the sponge samples. Sample abbreviations are presented in Figure 1.

**Figure S4.** Similarity of sponge-associated bacterial communities based on principal coordinate analysis (PCoA) of the pyrosequenced 16S rRNA genes. ­Group A includes samples N0, N2, D2, L2 and H2; group B includes samples N6, D6 and L6; and group C includes sample H6. Sample abbreviations are presented in Figure 1.

**Figure S5.** Phylogenetic tree (maximum likelihood) showing the location of the unclassified *Ectothiorhodospiraceae* (indicated by a black circle) in this study and the closest relatives in the NCBI database. The unclassified *Ectothiorhodospiraceae* identified in this study together with the uncultured clones from the sponges (cluster I and II) constitute a sponge-specific cluster.
